# Genetic diversity, phylogeography, and maternal origin of yak (*Bos grunniens*)

**DOI:** 10.1186/s12864-024-10378-z

**Published:** 2024-05-15

**Authors:** Xingdong Wang, Jie Pei, Lin Xiong, Pengjia Bao, Min Chu, Xiaoming Ma, Yongfu La, Chunnian Liang, Ping Yan, Xian Guo

**Affiliations:** 1 Key Laboratory of Yak Breeding in Gansu Province, Lanzhou Institute of Husbandry and Pharmaceutical Sciences, Chinese Academy of Agricultural Sciences, Lanzhou, 730050 P.R. China; 2 Key Laboratory of Animal Genetics and Breeding on Tibetan Plateau, Ministry of Agriculture and Rural Affairs, Lanzhou, 730050 P.R. China

**Keywords:** Genetic diversity, Mitochondrial DNA, Phylogeography, Yak, Glacial refugia

## Abstract

**Background:**

There is no consensus as to the origin of the domestic yak (*Bos grunniens*). Previous studies on yak mitochondria mainly focused on mitochondrial displacement loop (D-loop), a region with low phylogenetic resolution. Here, we analyzed the entire mitochondrial genomes of 509 yaks to obtain greater phylogenetic resolution and a comprehensive picture of geographical diversity.

**Results:**

A total of 278 haplotypes were defined in 509 yaks from 21 yak breeds. Among them, 28 haplotypes were shared by different varieties, and 250 haplotypes were unique to specific varieties. The overall haplotype diversity and nucleotide diversity of yak were 0.979 ± 0.0039 and 0.00237 ± 0.00076, respectively. Phylogenetic tree and network analysis showed that yak had three highly differentiated genetic branches with high support rate. The differentiation time of clades I and II were about 0.4328 Ma, and the differentiation time of clades (I and II) and III were 0.5654 Ma. Yushu yak is shared by all haplogroups. Most (94.70%) of the genetic variation occurred within populations, and only 5.30% of the genetic variation occurred between populations. The classification showed that yaks and wild yaks were first clustered together, and yaks were clustered with American bison as a whole. Altitude had the highest impact on the distribution of yaks.

**Conclusions:**

Yaks have high genetic diversity and yak populations have experienced population expansion and lack obvious phylogeographic structure. During the glacial period, yaks had at least three or more glacial refugia.

**Supplementary Information:**

The online version contains supplementary material available at 10.1186/s12864-024-10378-z.

## Introduction

The Qinghai-Tibet Plateau (QTP), one of the largest and youngest plateaus in the world, was formed around 40 million years ago (Ma) following the collision of the Indian tectonic plate with the Asian plate through several uplift events [1]. A large number of endemic species have appeared in the QTP and adjacent areas [2], due to its unique ecological environment. Yak is one of the representative species of QTP. At present, there are about 17.5 million yaks in the world, of which 94.4% are distributed in China [3]. Contemporary highland pastoralists rely on the strength and hardiness of domestic yak for transportation across vast mountainous terrain and for supplies of milk, meat, fiber, and dung for fuel [4]. Yaks play a vital role in socioeconomic development, pasture ecosystem maintenance, and agricultural biodiversity conservation in the QTP region [5]. Hence, yaks are referred to as “all-round animals” [6]. Morphological data suggest that yaks outside China originated from the Chinese yak [7]. According to previous studies, yaks were first domesticated in Tibet [8]. The combined archaeological and mitochondrial DNA (mtDNA) evidence suggests that Qinghai is one of the places where the yak either originated or was domesticated [7, 9, 10]. Qiu et al. re-sequenced the whole yak genome to find that the domestication of yak occurred 7 300 years ago [11]. However, the available genetic data do not provide a definitive conclusion and it is not known whether yak domestication occurred as a single event or multiple events in a single wild gene pool [7].

Domestication of animals is one of the major achievements of human civilization [12], although there had been many studies on yak ancestry, origin, and domestication, the answers to these questions are not clear. Studies have suggested an association between yak fossils and early human activity in Tibet [13], suggesting that yaks were first domesticated in Tibet [8]. The combined archaeological and mtDNA evidence suggests that Qinghai is one of the places where the yak either originated or was domesticated [7, 9, 10]. Qiu et al. re-sequenced the whole yak genome to find that the domestication of yak occurred 7 300 years ago [11]. However, the available genetic data do not provide a definitive conclusion and it is not known whether yak domestication occurred as a single event or multiple events in a single wild gene pool [7]. mtDNA is a good molecular marker for studying animal origins, evolution, classification, and population genetic diversity [14]. In recent years, the mtDNA D-Loop region sequence has been widely used to evaluate the origin, domestication, and genetic diversity of yaks [15, 16], cytochrome b (*Cytb*) is also a commonly used marker gene for studying the molecular genetic diversity of populations. Qi et al. [17] conducted a cluster analysis of the mtDNA D-Loop region and *Cytb* gene of 428 yak individuals from 29 yak populations in China and its surrounding countries. Phylogenetic analysis showed that 29 yak populations were clustered into three categories. In addition to mtDNA D-Loop region and *Cytb*, other regions can also be used for genetic diversity and phylogenetic analysis. Zhao et al. [18] determined the mtDNA Cytochrome c oxidase polypeptide III (*COIII*) sequence of 111 yak individuals from 11 yak populations in Tibet, indicating that Tibetan yaks have rich genetic diversity. Recently, researchers have discussed the importance of conducting complete mtDNA sequencing, because such analysis can produce detailed genetic maps when the sample size is large enough [19]. Wang et al. [10] sequenced the whole mitochondrial genome of yak, and used the 10 710 bp protein coding sequence except NADH dehydrogenase6 (*ND6*) for phylogenetic analysis. It was found that wild yaks were divided into three categories and domestic yaks were divided into two categories. It is speculated that there may be three maternal origins of wild yaks and two maternal origins of domestic yaks. However, most studies to date have been limited to the mitochondrial D-loop region and *Cytb* gene and have failed to clearly distinguish some important ancient clades in the domestic yak [15, 20, 21], the D-loop region is highly variable and information-rich in determining intraspecific diversity but often has parallel mutations [22, 23]. Recent studies have emphasized the importance of complete mtDNA sequencing [19], as this allows the investigation of 18 times as many sites as the D-loop region [10], and more detailed information can be obtained.

**Fig. 1:**
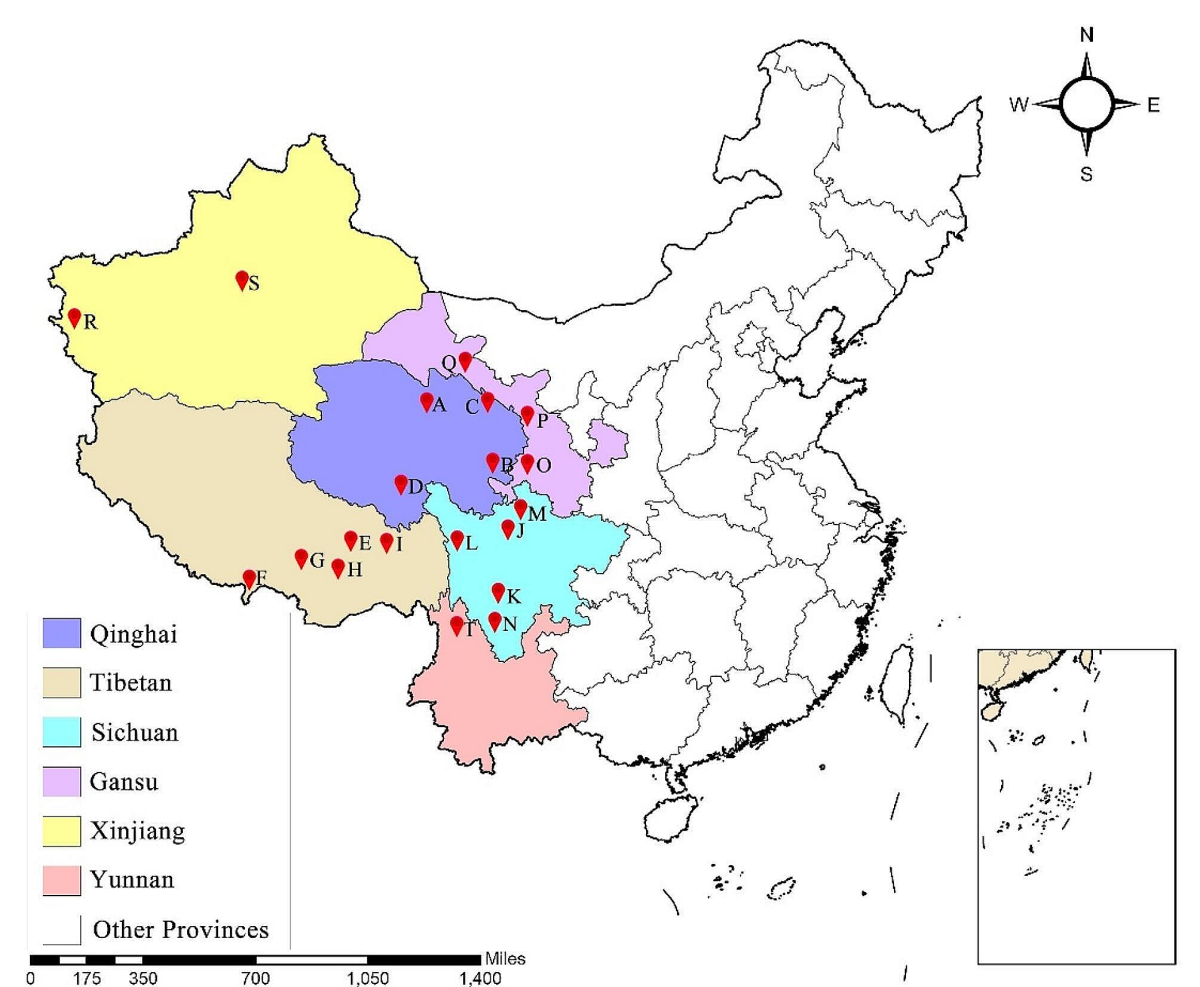
Collection locations of experimental samples. The details of the yak population represented at each sampling site are provided in Supplementary Table S1

At present, studies using the complete mitochondrial genome of the yak have mainly focused on a single yak genetic resource [24–26]. Also, there are few studies on the genetic evolution of the yak that have used the complete mitochondrial genome. Wild yaks that share a common ancestor with domestic yaks are still in existence, making the yak an excellent model species for studying the domestication of large animals. In our previous studies [27, 28] and here, we collected samples of genetic resources from every yak breed (Fig. 1) in China to ensure a large sample size for a detailed genetic map. The genetic diversity and phylogenetic structure of the yak were analyzed by the sequencing of the complete mitochondrial genome sequencing of these samples, which also provides a foundation for the effective protection and utilization of yak genetic resources.

## Results

### Identification and analysis of haplotypes

In total, 278 haplotypes were defined from 509 yaks. Of these, 28 haplotypes were shared by different breeds, and 250 haplotypes were unique to a specific breed. Among the 278 haplotypes, the H8 haplotype was the most common (found 68 times) and shared by 18 yak breeds except wild yak, Tianzhu white yak, and Pamir yak. In total, 23 haplotypes were identified in 25 wild yaks, of which only the H28 haplotype was shared by the wild yak and Changtai yak; the other 22 haplotypes were unique to wild yaks. In total, 11 haplotypes were defined in Tianzhu white yak; the H20 haplotype was shared by the Tianzhu white, Changtai, Huanhu, and Qinghai Plateau yaks, while the other 10 haplotypes were unique to the Tianzhu white yak. Twenty-two haplotypes were defined in 25 Pamir yaks; only the H150 haplotype was shared by the Pamir yak and Sibu yak, while the other 21 haplotypes were unique to the Pamir yak. Wild yak (96.65%), Pamir yak (95.45%), Tianzhu white yak (90.91%), Jiulong yak (89.47%), Yushu yak (81.25%), and Xueduo yak (80.95%) exhibited higher specific proportions of haplotypes (Table 1); for detailed information please see Supplementary Table S2.

**Table 1:** Haplotype statistics of yak breeds/populations

Breed	*N*	H	Specific haplotypes	Specific/Total (Haplotype)
Wild yak	25	23	22	96.65%
Huanhu yak	22	13	8	66.67%
Qinghai Plateau yak	22	16	9	56.25%
Xueduo yak	23	21	17	80.95%
Yushu yak	20	16	13	81.25%
Leiwuqi yak	25	13	7	53.85%
Niangya yak	25	22	13	59.09%
Pali yak	25	17	9	52.94%
Sibu yak	26	17	12	70.58%
Tibet Gaoshan yak	25	21	16	76.19%
Changtai yak	25	20	10	50.00%
Jinchuan yak	25	14	8	57.14%
Jiulong yak	25	19	17	89.47%
Muli yak	25	9	5	55.56%
Maiwa yak	25	13	8	61.54%
Gannan yak	25	17	11	64.71%
Tianzhu white yak	23	11	10	90.91%
Sunan yak	22	21	16	76.19%
Bazhou yak	25	18	12	66.67%
Pamir yak	25	22	21	95.45%
Zhongdian yak	26	11	6	54.55%
Total	509	278	250	89.93%

N, number of yaks; H, Number of haplotypes

### Phylogenetic analysis of yaks

**Fig. 2:**
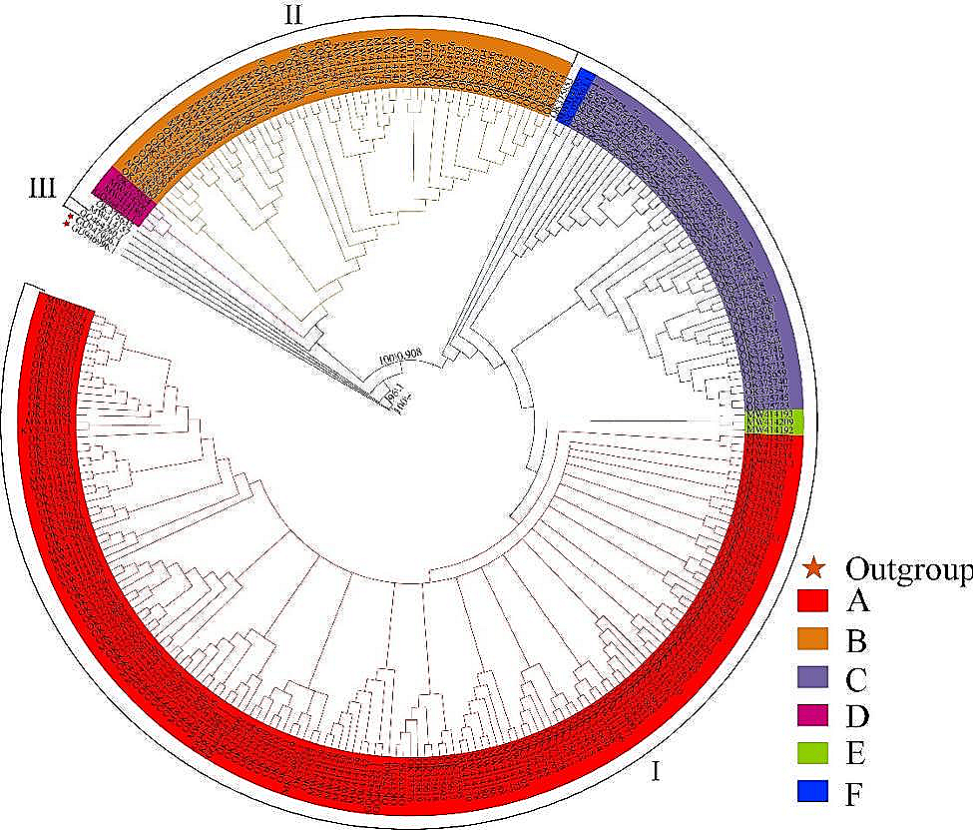
Phylogenetic tree of 278 haplotypes in the yak. Clades I, II, and III represent the three clades of yak. A, B, C, D, E, and F represent the six haplogroups of yak

Phylogenetic analysis was performed using 278 haplotypes (with *Bison bison* [GU947006.1, GU946996.1] as outgroups). The basic topological structures of the maximum likelihood (ML) and MrBayes phylogenetic trees were the same, showing three highly differentiated genetic clades with high support rates (Fig. 2). Most of the haplotypes were distributed within clades I and II. The yak phylogenetic tree contained six haplogroups (A-F) forming two clades, with clade I containing haplogroups A, C, E, and F and clade II containing haplogroups B and D; A-C were the three main haplogroups. Haplogroup A included all the yak populations, while haplogroup B included all populations except Tianzhu white yak and haplogroup C included all populations except Qinghai plateau yak, Tianzhu white yak, and Pamir yak. Haplogroups D-F contained only a few yak breeds. Among which the haplogroup D only contained the Yushu yak, wild yak, Sunan yak and Pamir yak; Haplogroup E only contained the Yushu yak; Haplogroup F only contained the Yushu yak and wild yak. Among all yak populations, the Yushu yak was common to all haplogroups.

**Fig. 3:**
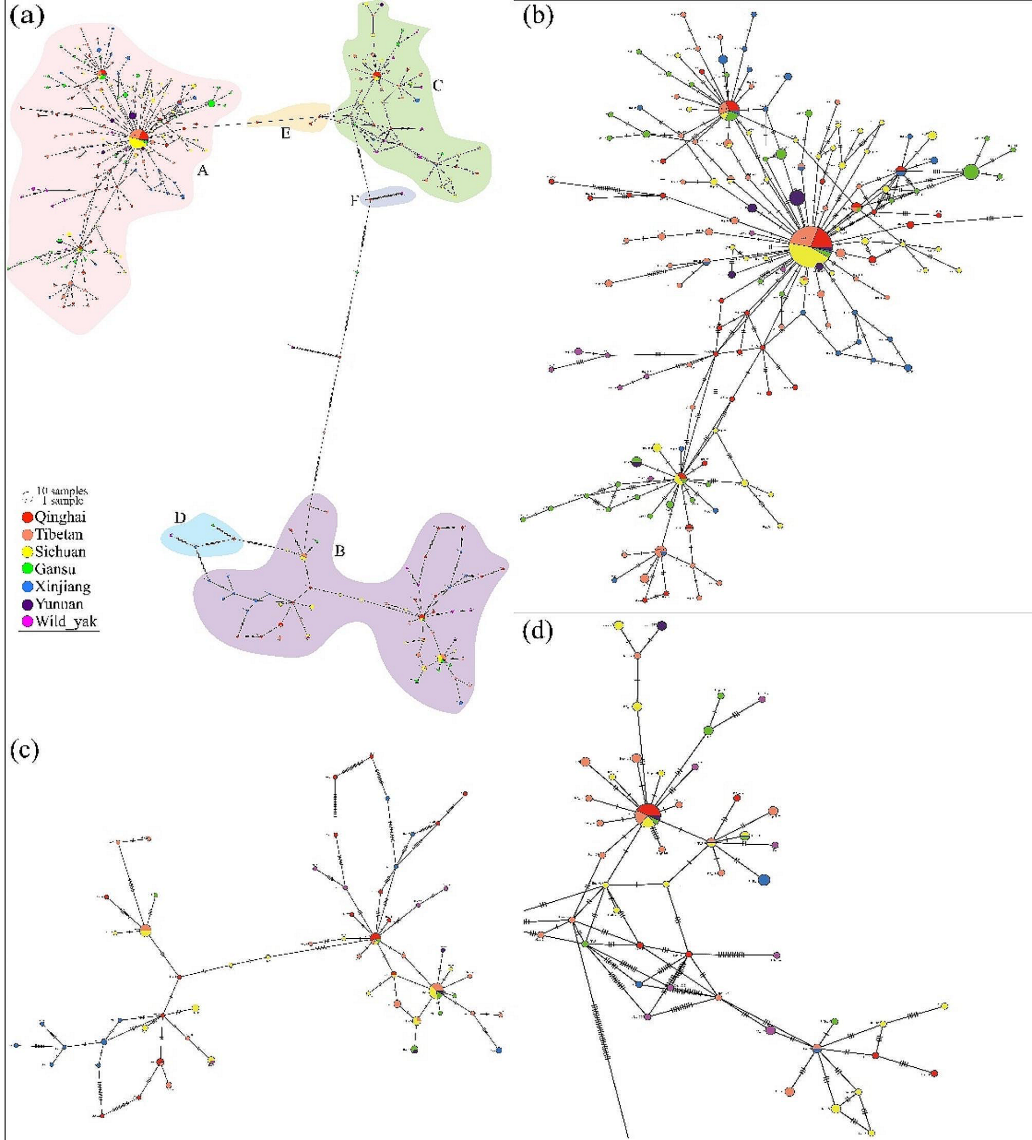
Network diagram of haplotypes. (**a**) The total network of mtDNA of 509 individuals, with different colors indicating yaks from different provinces. The network diagram of haplogroups (**b**) A, (**c**) B, and (**d**) C

Combined with the geographical distribution of yak populations, a haplotype network map was constructed based on yak mtDNA (Fig. 3). Consistent with the phylogenetic tree, the haplotype network diagram also revealed that yaks were divided into three clades and six haplogroups. The three main haplogroups A-C were distributed in a star-shaped radial pattern; the H8 and H36 haplotypes were shared by multiple individuals and located in the center of the star-shaped radial. The yaks in haplogroup A were distributed in all taxa. The yaks in haplogroup B were distributed in all yak distribution areas except Tianzhu in Gansu. The yaks in haplogroup C were distributed in all yak distribution areas except the Pamir region, Tianzhu in Gansu, and parts of Qinghai. Only the wild and Yushu yaks were widely distributed with the Yushu yak distributed in all haplogroups.

### Genetic diversity analysis of yak mtDNA

Higher haplotype diversity (*H*d) and nucleotide diversity (*P*i) values are indicative of greater genetic diversity in a population. Genetic diversity analysis showed that the haplotype and nucleotide diversities of the complete mitochondrial genome sequences of 509 individuals from 21 yak breeds/populations were 0.979 ± 0.0039 and 0.00237 ± 0.00076, respectively, indicating a high overall genetic diversity in the yak. The haplotype diversity of wild yak (0.993 ± 0.013), Sunan yak (0.996 ± 0.015), Pamir yak (0.990 ± 0.014), and Xueduo yak (0.992 ± 0.015) was relatively high, while Muli yak (0.810 ± 0.063) and Tianzhu white yak (0.830 ± 0.068) exhibited the lowest haplotype diversity. The nucleotide diversity of wild yak (0.00352 ± 0.00145), Leiwuqi yak (0.00319 ± 0.00065), Pamir yak (0.00309 ± 0.00018), and Tibet Gaoshan yak (0.00309 ± 0.00067) was relatively high; the lowest nucleotide diversity was of Tianzhu white yak (0.00034 ± 0.00013). The comprehensive analysis of haplotype and nucleotide diversities revealed that the genetic diversity of the wild yak was the highest, and that of the Tianzhu white yak was the lowest. Additional details including variable loci (*S*), *H*d, and *P*i are shown in Table 2.

**Table 2:** Genetic structure and diversity of yaks

Breed (yak)	Haplogroup						S	Hd ± SD	Pi ± SD
	A	B	C	D	E	F			
Wild	8	5	7	1	0	1	277	0.993 ± 0.013	0.00352 ± 0.00145
Huanhu	8	2	3	0	0	0	115	0.913 ± 0.045	0.00213 ± 0.00066
Qinghai Plateau	10	6	0	0	0	0	105	0.948 ± 0.036	0.00273 ± 0.00043
Xueduo	13	4	3	0	0	0	122	0.992 ± 0.015	0.00232 ± 0.00051
Yushu	5	5	1	1	3	1	115	0.963 ± 0.033	0.00276 ± 0.00047
Leiwuqi	3	5	4	0	0	0	121	0.883 ± 0.051	0.00319 ± 0.00065
Niangya	14	2	6	0	0	0	133	0.987 ± 0.017	0.00141 ± 0.00071
Pali	9	2	6	0	0	0	115	0.967 ± 0.019	0.00209 ± 0.00062
Sibu	13	2	2	0	0	0	126	0.951 ± 0.027	0.00125 ± 0.00066
Tibet Gaoshan	13	7	1	0	0	0	125	0.987 ± 0.015	0.00309 ± 0.00067
Changtai	14	3	2	0	0	0	133	0.980 ± 0.017	0.00196 ± 0.00071
Jinchuan	6	3	5	0	0	0	132	0.870 ± 0.061	0.00205 ± 0.00070
Jiulong	12	4	3	0	0	0	127	0.977 ± 0.018	0.00292 ± 0.00068
Muli	3	3	3	0	0	0	111	0.810 ± 0.063	0.00293 ± 0.00060
Maiwa	6	3	4	0	0	0	111	0.887 ± 0.051	0.00192 ± 0.00060
Gannan	10	2	5	0	0	0	127	0.970 ± 0.018	0.00181 ± 0.00068
Tianzhu	11	0	0	0	0	0	21	0.830 ± 0.068	0.00034 ± 0.00013
Sunan	14	4	1	1	0	0	134	0.996 ± 0.015	0.00285 ± 0.00076
Bazhou	15	1	2	0	0	0	139	0.970 ± 0.020	0.00148 ± 0.00074
Pamir	11	10	0	1	0	0	105	0.990 ± 0.014	0.00309 ± 0.00018
Zhongdian	6	3	2	0	0	0	124	0.865 ± 0.054	0.00162 ± 0.00065
Total	204	76	60	4	3	2	443	0.978 ± 0.0039	0.00237 ± 0.00076

*H*d, haplotype diversity; *P*i, nucleotide diversity; *S*, number of variable sites; SD, standard deviation

### Population dynamics analysis

By calculating the historical population dynamics of each haplogroup of yak (Table 3), the Tajima’s *D* value (-1.230) of the Total group was less than 0, and *P* > 0.05. Fu and Li’s *D* test (-7.262 (*P* < 0.05)) and Fu and Li’s *F* test (-4.543 (*P* < 0.05)) showed that the neutral test results of the Total group were contradictory. The mismatch analysis showed that the SSD and *H*_rag_ values of the yaks in the Total group were 0.0084 (*P* > 0.05) and 0.0038 (*P* > 0.05), respectively, this results indicated that the yak population had experienced the expansion. In haplogroups A and C, the results of neutral test showed *P* < 0.05, and the results of mismatch distribution showed *P* > 0.05, indicating that haplogroups A and C had experienced population expansion. In the haplogroup B, the results of the neutral test were *P* > 0.05, and the results of the mismatch distribution were *P* > 0.05, resulting in a contradiction in the calculation results of the historical population dynamics of the haplogroup B. The historical population dynamics of yaks (Supplementary Fig. S1) were analyzed by Bayesian Skyline Plot (BSP), revealing that each haplogroup of yaks experienced large-scale expansion. Due to the small number of individuals in the haplogroups D, E and F, the neutrality test and mismatch distribution analysis were not performed. We used the PermutCpSSR-2.0 software to analyze the *N*_*st*_ and *G*_*st*_ values at the population level. The results showed that *N*_st_ = 0.05225 > *G*_st_ = 0.03993 (*P* > 0.05). Analysis of Molecular Variance (AMOVA) analysis of the yak whole-mtDNA genomes showed that most (94.70%) of the genetic variation in yaks occurred within populations with only 5.30% observed between populations. This indicated the lack of obvious phylogeographical structure in yaks.

**Table 3:** Analysis of historical population dynamics

Haplogroup	Tajima’ D	Fu and Li’s D	Fu and Li’s F	S_sd_	*R*_ag_
A	-2.52791 (*P* < 0.01)	-5.57084 (*P* < 0.05)	-4.76865 (*P* < 0.05)	0.00216799 (*P* > 0.05)	0.00549254 (*P* > 0.05)
B	-1.56168 (*P* > 0.05)	-1.09486 (*P* > 0.05)	-1.55425 (*P* > 0.05)	0.00233210 (*P* > 0.05)	0.00307148 (*P* > 0.05)
C	-1.98563 (*P* < 0.05)	-3.39412 (*P* < 0.05)	-3.39233 (*P* < 0.05)	0.00389385 (*P* > 0.05)	0.00832564 (*P* > 0.05)
Total	-1.22978 (*P* > 0.05)	-7.26174 (*P* < 0.05)	-4.54348 (*P* < 0.05)	0.00842453 (*P* > 0.05)	0.00380330 (*P* > 0.05)

A, B and C denotes haplogroups A, B and C, respectively. Total represents the integration of all yak populations into a whole

### Estimation of differentiation time

Based on the Bayesian method, the differentiation time of the respective yak clades was calculated. The differentiation time of the two major clades (I and II) was about 0.4328 Ma with a 95% highest posterior density (HPD) of 0.3218–0.5326 Ma. The differentiation time of clades I and II and clade III was about 0.5654 Ma, with a 95% HPD of 0.4283–0.7162 Ma.

### Taxonomic status of the yak in the Bovidae

**Fig. 4:**
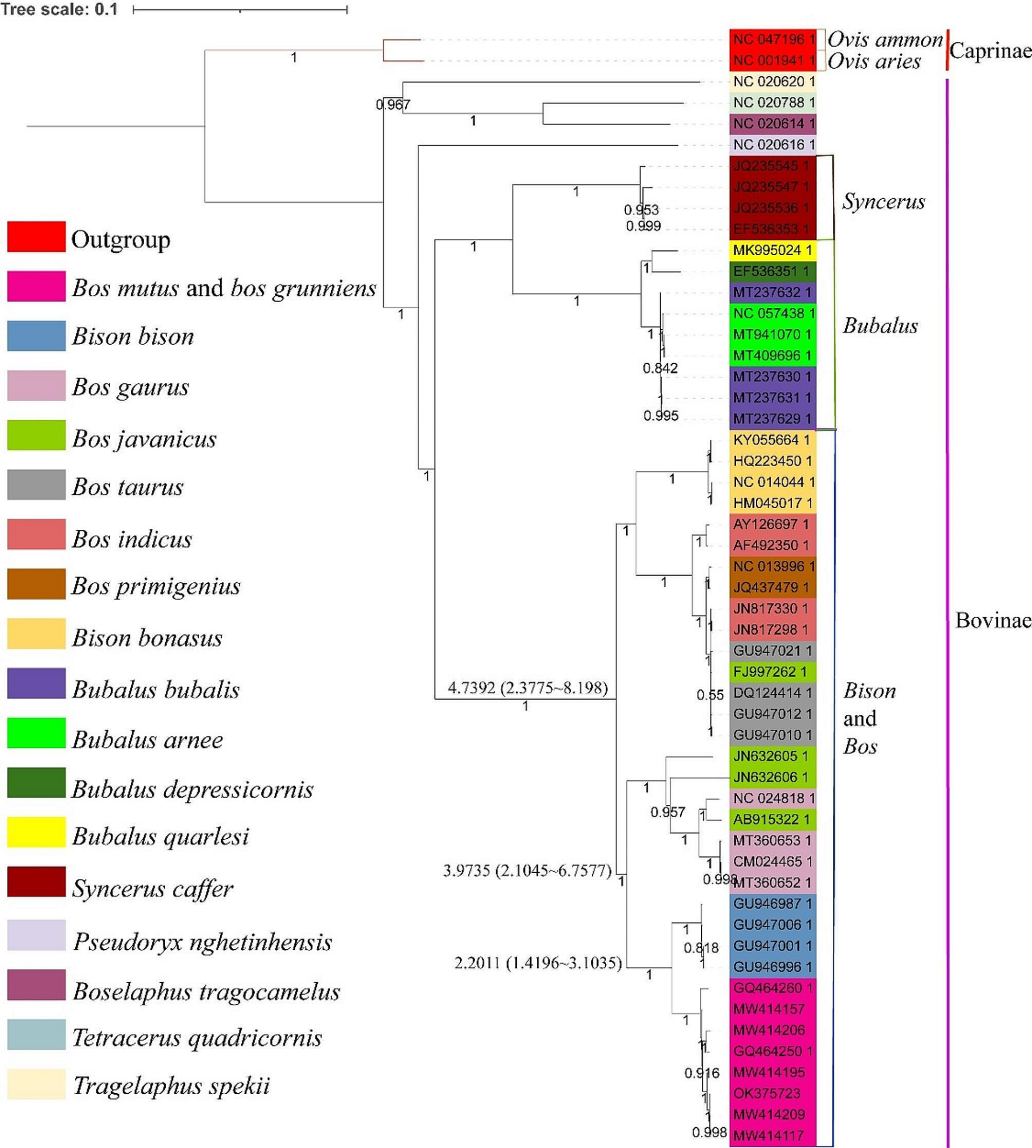
Taxonomic status of the yak in the Bovidae family

The Caprinae, including *Ovis ammon* (NC_047196.1) and *Ovis aries* (NC_001941.1), were selected as an outgroup for phylogenetic analysis. The results showed that *Bos grunniens* and *Bos mutus* were located together, and yaks as a whole clustered with *Bison bison*. The overall clustering relationship is shown in Fig. 4: (((((((*Bos grunniens + Bos mutus*) + *Bison bison*) + (*Bos gaurus + Bos javanicus*)) + ((*Bos taurus + Bos primigenius + Bos indicus*) + *Bison bonasus*)) + (((*Bubalus bubalis + Bubalus arnee*) + (*Bubalus depressicornis + Bubalus quarlesi*)) + (*Syncerus caffer*)) + *Pseudoryx nghetinhensis*) + ((*Boselaphus tragocamelus + Tetracerus quadricornis*) + *Tragelaphus spekii*)) + (*Ovis ammon* + *Ovis aries*)). Based on the differentiation time of the respective Bovidae species, the differentiation time of *Bos grunniens* and *Bison bison* was 2.2011 Ma, the differentiation time of *Bos grunniens, Bos gaurus*, and *Bos javanicus* was 3.9735 Ma, and the differentiation time of *Bos grunniens* and *Bos taurus* was 4.7392 Ma.

### Population distribution dynamics of the yak

**Fig. 5:**
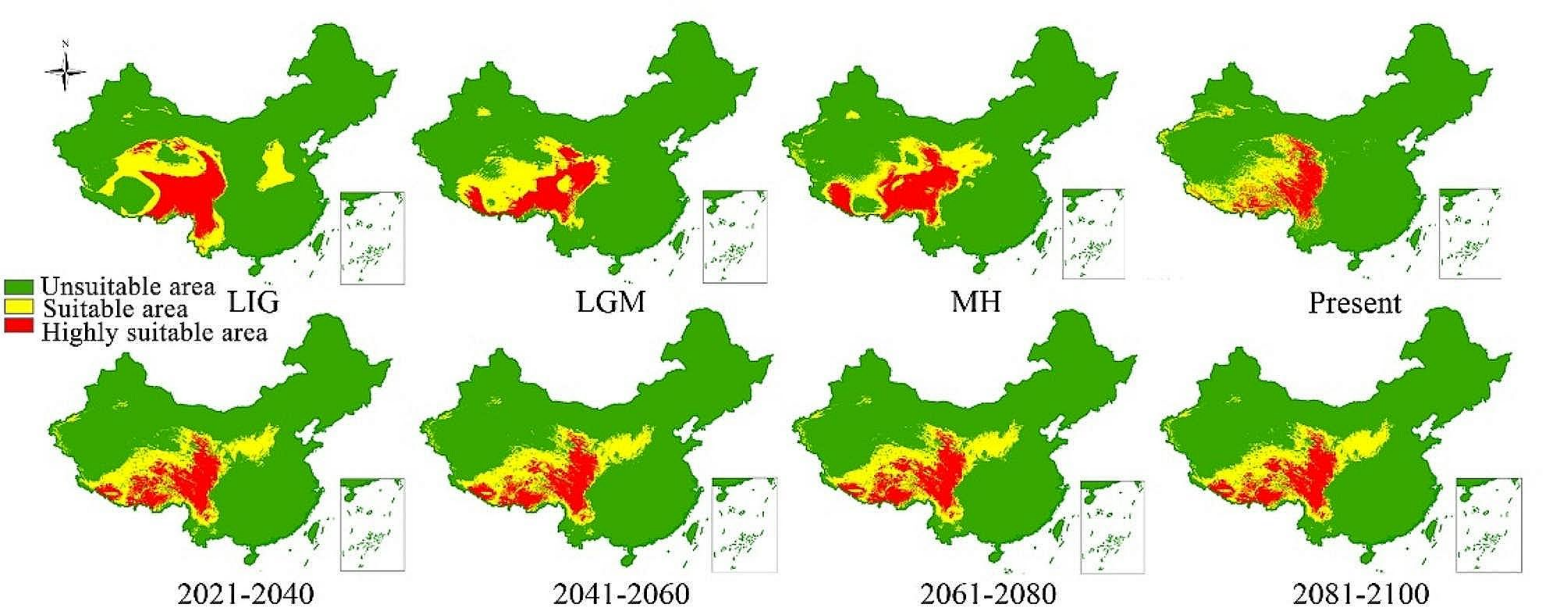
Dynamic distribution of yaks from the last interglacial period to 2100. LIG: last interglacial. LGM: last glacial maximum. MH: Mid-Holocene

The dynamic distribution of the yak in different periods was simulated by the MaxEnt model based on 11 selected environmental factors. Similar average Area under the curves (AUCs) of training (0.918) and test (0.873) data in different periods, indicated high accuracy of MaxEnt model simulation (Supplementary Table S3). The contribution rate of each environmental factor was tested by the knife-cut method. Elevation (74.6%) and annual temperature range (0.0009%) contributed the highest and lowest, respectively (Supplementary Table S4). A dynamic distribution map of yaks from the last interglacial (LIG) to 2100 was constructed (Fig. 5), showing that yaks were mainly located at the edge of the QTP during the last glacial maximum (LGM).

## Discussion

In the neutral test, values of Tajima’s *D* > 0 and *P* < 0.05 indicated that the population underwent a bottleneck effect and equilibrium selection, while Tajima’s *D* < 0 and *P* < 0.05 indicated that the population experienced the expansion and directional selection [29]. Fu and Li’s D*&F* indices can be used for neutral test [30]. However, the historical dynamics of the population cannot be inferred only by the Tajima’s *D* value, which is usually analyzed by a combination of neutral test and mismatch distribution. In this experiment, the neutral test results of the Total group were contradictory, and the mismatch analysis showed that the Total group experienced population expansion. In haplogroup B, the neutral test results are contrary to the mismatch distribution results. Only when the neutral test was negative and *P* < 0.05, it indicated that the population was significantly deviated from the neutral mutation, indicating that it was intervened by artificial or natural selection, and the curve of mismatch distribution analysis was unimodal distribution, indicating that the population was in an expanding state. Due to the neutral test results of Total group and haplogroup B are contradictory and the neutral test results are contrary to the mismatch distribution results, which makes it difficult to judge whether the population has expanded during evolution. The result of mismatch distribution is based on the ideal state, which is limited in practical applications. In fact, the historical population dynamics are often more complex than the parameter models involved in these methods. The BSP method is based on the clustering theory and is used to quantify the relationship between gene sequence lineages and population geographic history. Using molecular clock or fossil correction, with the help of BEAST series software, the Markov Chain Monte Carlo (MCMC) algorithm based on Markov chain is used to calculate the change of effective population size with time. Especially for the analysis of different genes and a small number of individuals, this method can better estimate the effective population size. Therefore, the neutral test results deviate from 0 and *P* < 0.05 is only a prerequisite. If the neutral test results are inconsistent with the BSP results, the BSP results (Supplementary Fig. S1) should be used.

Genetic variation in yaks originated largely within populations without any obvious geographical structure. A study in goats also showed no obvious phylogeographical structure between the highly differentiated genetic clades and haplogroups due to the large-scale migration of goats around the world [31]. Notably, the domestication of yaks started 7300 years ago [11]. After domestication, yaks migrated with herdsmen on a large scale, enabling integration with yak populations in distant geographical regions which would account for the lack of an obvious pedigree geographical structure in yaks.

Genetic diversity is a central facet of biological diversity [32]. High *H*d and *P*i values indicated high genetic diversity in wild, Pamir, Xueduo, Qinghai Plateau, Yushu, Tibet Gaoshan, Sunan, and Jiulong yaks. The Gannan, Niangya, Bazhou, and Sibu yaks had high *H*d but low *P*i values. While a single base mutation can generate a new haplotype, this has little impact on nucleotide diversity. Compared with haplotype diversity, increases in nucleotide diversity take longer. Therefore, the bottleneck effect caused by repeated changes in the environment and the rapid population expansion and variation accumulation after the bottleneck effect can lead to high *H*d and low *P*i values [33]. The low *H*d and high *P*i values of Leiwuqi and Muli yaks can be attributed to selective pressure from a new environment after migration or the coming into contact of two relatively independent populations or simply to an insufficient number of samples [33]. This requires further analysis. Zhongdian and Tianzhu white yaks exhibited low *H*d and *P*i values, indicating the influence of the founder effect, i.e., the re-establishment of a new population from a few individuals. The numbers in this population would increase but without an increase in genetic diversity [33]. Low levels of genetic diversity lead to inbreeding and decreased population fitness [34]. Which is consistent with the artificial selection process seen in the Tianzhu white yak. For 130 years, the Tianzhu white yak has been bred by strict selection of fur color [35], and this strict artificial selection and control have led to low gene flow between the Tianzhu white yak and other yak populations, resulting in a high degree of genetic differentiation, consistent with the earlier findings that the Tianzhu white yak differed from other domestic yak populations [11].

Previous studies based on mtDNA D-loop fragments showed that yaks were divided into two clades (I and II), with a differentiation time of 100 000–130 000 years ago [7]. The differentiation time of the main clades in the yak phylogenetic tree was also calculated based on the third codon of the protein-coding gene of mtDNA. This new method and new fossil marker (2.5 Ma) revealed the differentiation time of three yak clades to be between 420 000 and 580 000 years ago [10]. Analysis of the whole mtDNA also revealed three main clades in the yak evolutionary tree. Differentiation between clades I and II occurred 0.4328 Ma with a 95% HPD of 0.3218–0.5326 Ma. Differentiation between clade III and clades (I, II) occurred before 0.5654 Ma with a 95% HPD of 0.4283–0.7162 Ma. This is in agreement with previous findings [10]. In addition, our estimated differentiation time is also consistent with the records of glacial events in the middle and late Pleistocene in the Tibetan Plateau [36], suggesting that glacial activity in the middle and late Pleistocene may have triggered yak migration and therefore genetic differentiation.

There have been three great glacial epochs in the evolutionary history of the earth. The Quaternary glacial epoch was the most recent in geological history and had a major impact on modern biology [37]. Due to specific buffering environmental characteristics, the glacial refugia contained unique genetic lineages during a series of climatic fluctuations occurring during the Tertiary and Quaternary epochs [38], allowing animals and plants to escape from the harsh climatic conditions of the glacial epoch [39]. Glacial refuges are the starting point for the post-glacial redistribution of species after deglaciation [40]. Large glacial refugia may last for hundreds of thousands of years or even longer, and, therefore, the isolation of glacial refugia may have accelerated the differentiation of a species’ populations [41]. The Hengduan Mountains in the southeastern part of the QTP rose rapidly between the Late Miocene and Late Pliocene [42]. The QTP was never completely covered by glaciers during the Quaternary [43] and is one of the most important biodiversity research hotspots in the world [44]. The QTP also formed an important refuge and place of origin for species during the glacial epoch, resulting in rich species diversity and unique geological characteristics [45, 46]. Studies on *Juniperus przewalskii* [47], *Metagentiana striata* [48], and *Pedicularis longiflora* [49] indicated that some species may have retreated to refuges on the edge of the QTP during the glacial epoch and recolonized the plateau and adjacent highlands at the end of epoch. In addition, some glacial refugia on the QTP supported the survival of plant species, such as *Hippophae rhamnoides* [50] and *Spiraea alpina* [51], during climate change [1, 52]. Research on *Aconitum gymnandrum* [53], *Hippophae tibetana* [54], *Rhodiola alsia* [55], and *Rhodiola chrysanthemifolia* [56] has shown that there were several miniature refugia on the QTP. The multiple glacier refuge scenario fits well with the hypothesis of multiregional and multiscale glaciation during the Pleistocene [49, 57]. The mtDNA phylogeny analysis of yak revealed three clades. Wang et al. [53] found four *Aconitum gymnandrum* populations that possibly evolved from four independent glacial refugia during the LGM. The single refuge hypothesis generally advocates a network of stellate haplotypes in the entire population [58]. The network diagram of the yak mitochondrial genome (Fig. 3) indicated at least three stellate haplotype network structures in the yak, suggesting at least three glacial refuges in the yak evolutionary history. The MaxEnt model was used to predict the dynamic distribution of yaks from the LIG to the present time (Fig. 5). Yaks were mainly but not entirely located on the edge of the QTP during the LGM, suggesting that the yak refuges during the glacial epoch were associated mainly with the marginal areas of the QTP. In *Alopex lagopus*, the population distribution did not change to follow available habitats during the post-glacial contraction phase but instead went extinct [59]. This suggests that arctic species did not respond to climate change in the same way as temperate species, i.e., their distribution ranges contracted rather than expanded during interglacial warming. It is still unclear whether species migrated to the refuge to cope with climate change or populations outside the refuges were directly made extinct [58]. With the lack of detailed information about some sampling points, yak fossil information, and other data, it is impossible to assess the location of glacial refugia during the glacial epoch, or whether yaks outside the glacial refugia underwent direct extinction.

The yak classification in the Bovidae is very different. Linnaeus placed the yak together with *Bos taurus* and *Bos indicus* in the *Bos* genus. Based on its morphological and skeletal differences from other cattle species, Gray, Olsen, and Geraads classified the yak into an independent yak genus [60]. This study explored the taxonomic status of yak from the direction of maternal inheritance. The representative mtDNA of yaks from three clades and six haplogroups were selected and those of other cattle were downloaded from the GenBank database to determine the phylogenetic relationships among bovids. Each species was represented by at least two individuals. The clustering results showed that the individuals of each species were first clustered into one category; the yaks as a whole were clustered together with *Bison bison*, and then with other cattle. This is consistent with a previous report [60] on the *Cytb* gene of domestic yak mtDNA and another report [61] on the mtDNA of wild yak. The time of separation between the yak and other bovid species was calculated by the Bayesian method, finding a value of 2.2011 Ma for the separation between yak and *Bison bison*, which was consistent with the phylogenetic tree structure. This study supports the classification of Li [60] and Zhong [61] who classified the yak into a separate genus that included two species, namely, domestic and wild yak.

## Conclusions

In conclusion, yaks generally showed high genetic diversity, among which wild yaks had the highest genetic diversity and Tianzhu white yaks had the lowest genetic diversity. The mtDNA genome-wide genetic variation of yak mainly occurs within the population and lacks obvious phylogeographic structure. Phylogenetic analysis revealed that yak had three highly differentiated genetic branches with high support rate. The differentiation time of clades I and II were about 0.4328 Ma, and the differentiation time of clades (I and II) and III were 0.5654 Ma. Yak contains 6 haplogroups. In all yak populations, Yushu yak is distributed in all haplogroups, and the three major haplogroups A-C are distributed in a star-shaped radial pattern. Yaks have experienced population expansion. At the last LGM, the yak’s glacial refugia was mainly located at the edge of QTP. The effects of altitude and annual temperature range on the dynamic distribution of yaks were the highest and lowest, respectively. The classification showed that yaks and wild yaks were first clustered together, and yaks were clustered with American bison as a whole.

### Methods

#### Animals and sample collection

The mitochondrial genomes of 372 yaks were sequenced. These yaks were from 15 breeds identified by the National Livestock and Poultry Genetic Resources Commission. Supplementary Table S1 lists information on the breed, numbers, mitochondrial genome accession numbers and geographical coordinates of the sampling points of the yaks. Blood samples were collected from 20 to 26 yaks from each breed/population. There was no sex restriction, and the yaks were three to eight years old without any genetic relationships. Blood samples were collected from the jugular veins of the yaks into EDTA anticoagulant tubes, immediately stored in the vehicle refrigerator, and transported to the laboratory within 24 h. The samples were then stored at -80 °C in the Key Laboratory of Yak Breeding Engineering of Gansu Province. The blood storage numbers were R-5-1-001, R-5-1-002, and R-5-1-003, and the DNA was extracted from the samples within one month. No yaks were sacrificed in this experiment, and all blood samples were taken from live yaks. The jugular vein area of the yaks was locally disinfected with alcohol before blood sample collection. After collected blood samples, wiped with iodine to prevent any possible wounds being infected.

### Extraction, amplification, and sequencing of the mitochondrial genome

The primers designed by Wang [10] and synthesized by Xi’an Qingke Biotechnology Co., Ltd. (Xi’an, China) were used to amplify the whole mitochondrial genome of the yaks. The reagents, methods, and reaction conditions used for the extraction and amplification of the mitochondrial genome were as previously described [27]. After amplification, the quality of the products was assessed using 1% agarose gel electrophoresis before sequencing at Xi’an Qingke Biotechnology Co., Ltd. (Xi’an, China).

### Analysis of genetic structure and genetic diversity

The complete mitochondrial genomes (accession numbers OK375501–OK375872) of the 372 yak individuals were sequenced. After the inclusion of the mitochondrial sequences obtained in an earlier study (accession numbers MW414100–MW414210, MK124955.1), a total of 484 domestic yak individuals were used for experimental analysis. This final sample set covered all morphological groups and distribution ranges of the domestic yaks. In addition, the complete mitochondrial genome sequences (accession numbers GQ464246.1–GQ464266.1, MK033130.1, KR106993.1, KY829451.1, NC_025563.1) of 25 wild yaks were downloaded from National Center for Biotechnology Information (NCBI), increasing the total number of yak mitochondrial complete genome sequences to 509.

MAFFT 7.0 was used for sequence alignment [62]. AMOVA were performed using Arlequin v 3.5 software to determine the level of differentiation among the yak populations; the number of permutations was set to 10 000 [63]. DNAsp 6.0 [64] software was used to calculate the *F*_st_ among populations, detect the degree of genetic differentiation among populations, and perform genetic diversity analysis and neutrality tests. PermutCpSSR-2.0 software was used to calculate the *N*_st_ and *G*_st_ values at the species level (parameters set to 1 000 substitutions) to explore the relationship between genetic and geographical distances and examine whether the species distribution had a genetic geographical structure [65]. The yak network diagram was constructed with Popart [66] software. Default settings were used for all the software not described in detail.

### Construction of phylogenetic tree

The yak phylogenetic tree was constructed with PhyloSuite [67]. The IQ-tree module was used to construct the ML phylogenetic tree. The optimal base replacement model was K3Pu + F + R5, “Bootstrap” was Standard, “Num of Bootstrap” was 1 000, and the SH-alRT test was enabled. The default value of repeat sampling times was 1 000. The Mbayes module was used for the construction of 4 MCMC chains of a Bayesian phylogenetic tree, and the optimal base replacement model was HKY + F + I + G4. The number of generations was 100 000 000, the sampling frequency was 1000, the number of runs was 2, and 25% of aging samples were discarded.

### Estimation of differentiation time

Beast v1.10 [68] was used to estimate the differentiation time, with a Bayesian Information Criterion optimal model of TIM2 + F + I + G4. The corrected Akaike Information Criterion optimal model was GTR + F + R5. According to the fossil time query website (http://www.timetree.org/), the point of divergence between *Bos mutus* and *Bison bison* was 1.5 (0.2–3.9) Ma; the relaxed molecular clock model from Beast v 1.10 was used to calculate the differentiation time. A total of 5 000 000 generations, obtaining a tree every 1 000 generations, and ESS > 200 were used.

### Research on population distribution dynamics

The climate data from all periods were downloaded from the WorldClimate database (http://www.worldclim.org/). Chinese soil data were obtained from the World Soil Database (HWSD) and downloaded from the National Ice Frozen Desert Science Data Center and the National Special Environment and Special Function Observation Research Station sharing service platform (http://www.crensed.ac.cn/portal/). The map data were downloaded from the National Basic Geographic Information Center (http://www.ngcc.cn/ngcc/). The mask method in ArcGIS 10.5 (https://developers.arcgis.com/) was used to extract the variable data from all environmental factors based on the map data and environmental data of China. MaxEnt v 3.4.1 (https://biodiversityinformatics.amnh.org/open_source/maxent/) was used to construct the distribution dynamics of species in different periods. AUC < 0.60 indicated the failure of the prediction model; AUC 0.60–0.70 indicated that the model prediction results are poor; AUC 0.70–0.80 indicated that the model prediction results are general; AUC 0.80–0.90 indicated that the model prediction results are accurate; AUC 0.90–1.00 indicated that the model prediction results are very accurate [69].

### Electronic supplementary material

Below is the link to the electronic supplementary material.

Supplementary Material 1

Supplementary Material 2

## Data Availability

The datasets generated and analyzed during the present study are available in the GenBank repository, under the accession number: OK375501–OK375872 (https://www.ncbi.nlm.nih.gov/genbank/).The data supporting the conclusions of this study are available in the supplementary table.

## Abbreviations

D-loop: Displacement loop
QTP: Qinghai-Tibet Plateau
Ma: Million years ago
mtDNA: Mitochondrial DNA
*Cytb*: Cytochrome b
Hd: haplotype diversity
Pi: Nucleotide diversity
HPD: Highest posterior density
LIG: Last interglacial
LGM: last glacial maximum
NCBI: National Center for Biotechnology Information
AUC: Area under the curves
