# Supplementary material for: Genetic diversity, phylogeography, and maternal origin of yak (Bos grunniens)
Source: BMC Genomics. 2024 May 15;25:481. doi: 10.1186/s12864-024-10378-z (PMC11097540; doi:10.1186/s12864-024-10378-z)
Supplement: Supplementary file 2 — Supplementary Material 2 [file 12864_2024_10378_MOESM2_ESM.pdf]

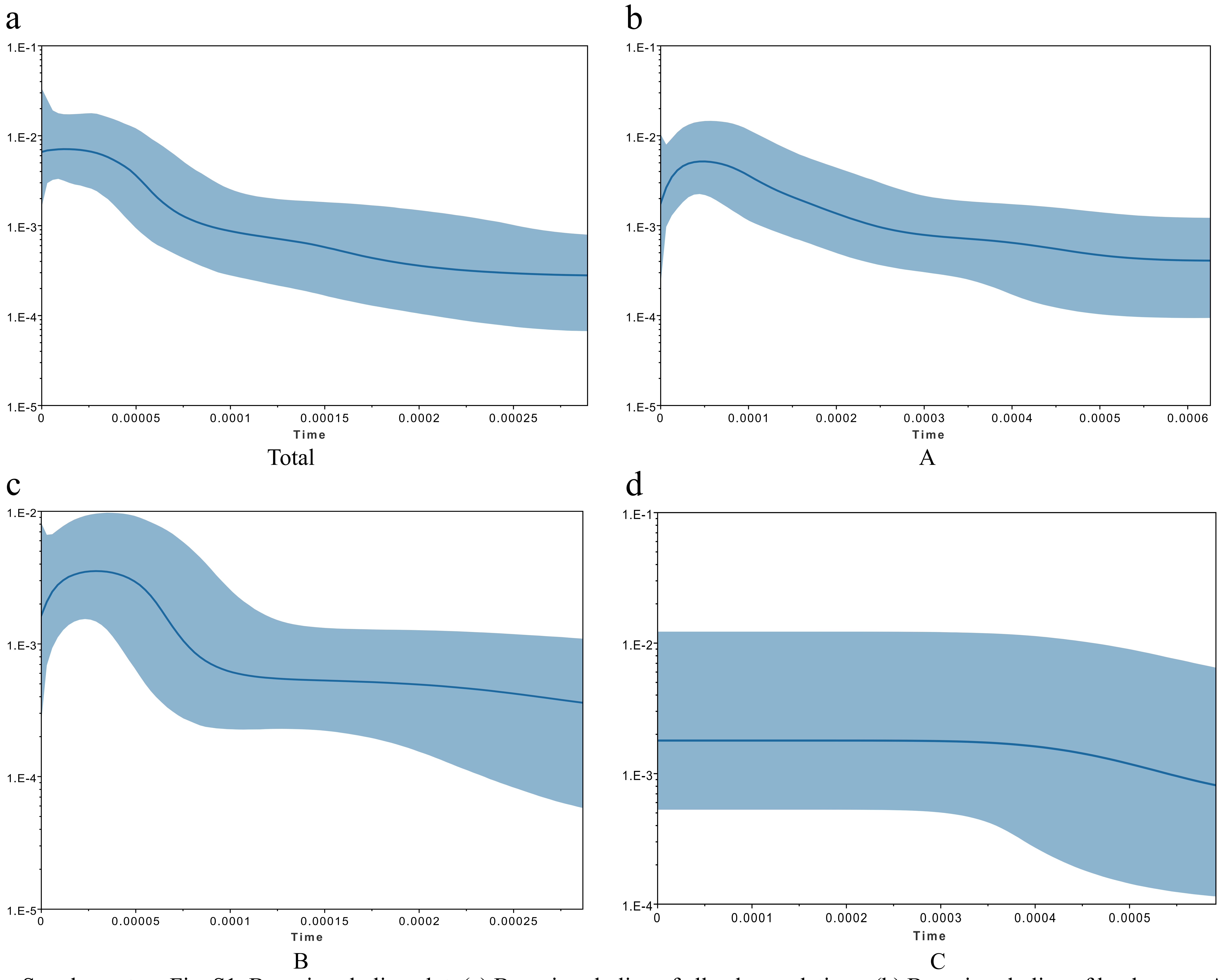

Supplementary Fig. S1. Bayesian skyline plot. (a) Bayesian skyline of all yak populations. (b) Bayesian skyline of haplogroup A. (c) Bayesian skyline of haplogroup B. (d) Bayesian skyline of haplogroup C.
